# Coffee intake and CYP1A2*1F genotype predict breast volume in young women: implications for breast cancer

**DOI:** 10.1038/sj.bjc.6604687

**Published:** 2008-09-23

**Authors:** H Jernström, M Henningson, U Johansson, H Olsson

**Affiliations:** 1 Department of Oncology, Clinical Sciences, Lund University, Barngatan 2B, SE-221 85 Lund, Sweden; 2 Faculty of Health and Society, Malmö University, SE-205 06 Malmö, Sweden; 3 Department of Oncology and Cancer Epidemiology, Clinical Sciences, Lund University, Barngatan 2B, SE-221 85 Lund, Sweden

**Keywords:** breast cancer, CYPIA2, coffee, breast volume, BRCA1/2, oral contraceptive

## Abstract

As breast volume may be associated with heart cancer risk, we studied the relationship between breast volume, CYP1A2*1F and coffee intake. Among healthy premenopausal non-hormone users, 3+ cups per day was associated with lower volume only in C-allele carriers (*P*_interaction_=0.02), which is consistent with reports that coffee protects only C-allele carriers against breast cancer.

A recent study reported that coffee reduced the risk of breast cancer in *BRCA1* carriers with the CYP1A2*1F C-allele but not in those with the CYP1A2*1F A/A genotype (Kotsopoulos *et al*, 2007). Another study found significantly lower coffee consumption in sporadic breast cancer cases with at least one C-allele compared with the A/A genotype (Bågeman *et al*, 2008) in spite of the fact that coffee consumption is similar across CYP1A2*1F genotypes in the general population (Cornelis *et al*, 2006). Some studies (Vatten *et al*, 1990; Baker *et al*, 2006; Nkondjock *et al*, 2006; Ganmaa *et al*, 2008), but not all (Rosenberg *et al*, 1985; Vatten *et al*, 1990; McLaughlin *et al*, 1992; Stensvold and Jacobsen, 1994; Michels *et al*, 2002), have reported that moderate-to-high coffee consumption protects against breast cancer, though a mechanism is unknown. None of these studies took CYP1A2*1F into account.

Coffee is metabolised by the CYP1A2 enzyme, which also plays a key role in oestrogen metabolism (Le Marchand *et al*, 1997; Lee *et al*, 2003). Coffee contains not only caffeine but also phytooestrogens that can interact with and even block the oestrogen receptor (Magee and Rowland, 2004). Women with high coffee intake and the highly inducible CYP1A2*1F A/A genotype (Sachse *et al*, 1999) have a high 2-hydroxyoestrone (2-OHE1) to 16*α*-OHE1 ratio (Jernström *et al*, 2003; Bradlow *et al*, 2006; Klug *et al*, 2006). 2-Hydroxyoestrone is a weak oestrogen (Schneider *et al*, 1984), whereas 16*α*-OHE1 is procarcinogenic (Telang *et al*, 1992). In line with this, we found that breast volume in breast cancer patients was significantly higher in women with a lower 2-OHE to 16*α*-OHE1 ratio (Klug *et al*, 2006). Coffee also affects testosterone and SHBG levels (Ferrini and Barrett-Connor, 1996; Nagata *et al*, 1998). Although hormonal regulation of breast tissue has been extensively studied, little is known about hormones, genes, diet influence, density, size and diseases of the breast.

Mammographic density is a strong risk factor for breast cancer (Boyd *et al*, 2005), the risk increasing with larger percentage density. The non-inducible CYP1A2*1F C-allele was associated with the percent density in normal-weight postmenopausal women not using hormone therapy (Takata *et al*, 2007). CYP1A2 enzyme activity has also been associated with density (Hong *et al*, 2004), and coffee induces CYP1A2 enzyme activity (Djordjevic *et al*, 2007). There is no direct correlation between breast density and size after adjustment for waist-to-hip ratio and BMI (Beijerinck *et al*, 1995). However, a larger breast size has been associated with increased breast cancer risk (Kato *et al*, 1995), especially in lean women (Kusano *et al*, 2006) or in women with proliferative breast disease (Dupont and Page, 1987). We therefore investigated whether coffee intake was associated with breast volume in young women and whether any association was modified by the CYP1A2*1F genotype.

## Materials and methods

A total of 269 young healthy Swedish women from breast cancer high-risk families volunteered to participate in this study between 1996 and 2006. The study population has previously been described in detail (Hietala *et al*, 2008). The Ethics Committee of Lund University approved the study. All women signed a written informed consent.

The questionnaire included questions on reproductive factors, the use of hormonal contraceptives, smoking, coffee consumption and so on. One large cup (approximately 300 ml) was counted as two traditional cups of coffee (approximately 150 ml). Blood samples and body measurements were collected both during menstrual cycle days 5–10 and days 18–23. Breast volumes were measured while the woman was on her hands and knees with the breasts hanging down. The volume was approximated to a pyramid (base area × height/3). Total breast volume, that is the sum of the right and left breast volumes, was calculated in each visit.

Mutation testing of the *BRCA1* and *BRCA2* genes was not performed as part of this study and carrier status was obtained from the clinical records as previously described (Hietala *et al*, 2008). CYP1A2*1F analysis (rs762551) PCR primers 5′-AGGTATCAGCAGAAAGCCAGCAC and 5′-GCTGAGGGTTGAGATGGAGACAT were used as described earlier (Bågeman *et al*, 2008). Thirty-eight samples were run in duplicate with 100% concordance.

Oestradiol was analysed with Elecsys system 1010/2010 in EDTA plasma. This system uses a competitive immunoassay with a polyclonal antibody directed towards 17-*β* oestradiol. Intra-assay variation ranged from 1.9 to 5.7% and the inter-assay variation varied from 2.3 to 6.2%.

Testosterone, SHBG and progesterone in EDTA plasma were measured by electrochemiluminescent immunoassay by Elecsys 1010/2010 Modular analytics E170 analyser with the Roche Elecsys 1010/2010 (Roche Diagnostics, Mannheim, Germany). The intra-assay variation was 2.5–6.8% for testosterone, 1.8–4.0% for SHBG and 1.8–4.8% for progesterone. Insulin-like growth factor (*IGF*)*-1* was analysed in EDTA plasma with a radioimmunoassay method at Karolinska Hospital, Stockholm, Sweden as described earlier (Bang *et al*, 1991; Jernström *et al*, 2005). *IGFBP-3* was analysed with the IMMULITE 2000 solid-phase enzyme-labelled chemiluminescent immunometric assay as described earlier (Jernström *et al*, 2006).

The statistical software programs SPSS13.0 and STATA 10.0 were used. Breast volumes and weights were not normally distributed and were transformed using the natural logarithm (ln). The CYP1A2*1F genotype was classified into two groups (A/A or any C-allele). An interaction term was calculated between CYP1A2*1F and coffee (3+ cups per day). Multivariate linear regression analyses were performed to evaluate the interaction between CYP1A2*1F and coffee on breast volume, and further adjusted for family clustering using the cluster option of the logistic command in STATA. Geometric means of standardised breast volumes and 95% prediction intervals were calculated. The breast volumes were standardised at age 29 years, weight (ln 67 kg), non-smoking and nulliparity. Multivariate logistic regression was used to analyse the association between coffee and hormone and growth factor levels. A *P*-value of <0.05 was taken to be significant. All *P*-values were two sided.

## Results

Characteristics of the 269 women and CYP1A2*1F genotypes are presented in Table 1. CYP1A2*1F were available for 267 of the 269 women. CYP1A2*1F did not differ according to *BRCA1/2* mutation status (Table 2). Coffee consumption did not significantly differ between the CYP1A2*1F after adjusting for age and smoking.

Total breast volumes during menstrual cycle days 18–23 were available for 255 women who were not currently breast-feeding and without previous breast surgeries. Age-adjusted coffee consumption was positively associated with current smoking (*P*<0.0001), but not with nulliparity or current hormonal contraception. Total breast volumes were positively associated with weight (*β*=2.119; *P*<0.0001), but not with age, current hormonal contraception, nulliparity, current smoking or CYP1A2*1F.

We stratified the women according to the use of hormonal contraceptives as this changes endogenous hormone levels (Jernström and Olsson, 1994; Jernström *et al*, 1997, 2005), influences breast volumes in nulliparous women (Jernström and Olsson, 1997) and reduces the CYP1A2 enzyme activity (Rietveld *et al*, 1984).

**Figure 1:**
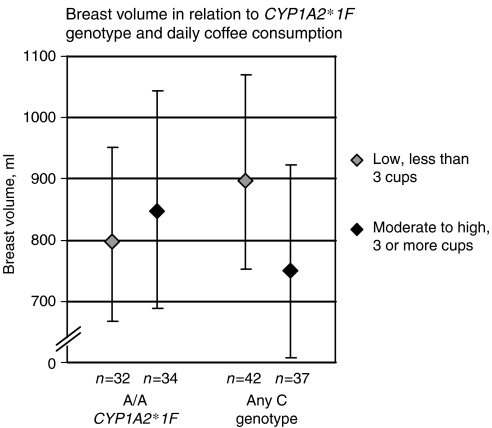
The figure shows the geometric means of the breast volumes in women according to their CYP1A2*1F genotype and coffee consumption. The breast volumes were standardised at age 29 years, body weight ln 67 kg, nulliparity and non-smoking. Ninty-five percent prediction intervals are presented. The interaction was significant (*P*=0.02). Please note the broken *y* axis.

Among the non-users, of whom 145 had available breast volumes and CYP1A2*1F genotypes, the association between a moderate-to-high coffee consumption (3+ cups per day) and breast volume was significantly modified by CYP1A2*1F genotype. Among women carrying at least one C-allele, moderate-to-high consumption was associated with lower standardised breast volumes compared with low consumption (896 *vs* 749 ml) whereas the standardised volumes were somewhat larger in women with moderate-to-high coffee consumption and the A/A genotype (797 *vs* 847 ml) (*β*=−0.303; *P*_interaction_=0.02) (Figure 1). The interaction remained significant after further adjustment for *BRCA1/2* mutation status (*β*=−0.304; *P*=0.02) and after exclusion of women who currently smoked or used snuff (*β*=−0.328; *P*=0.03). The women belonged to 105 different families. The interaction remained significant after adjustment for family clustering (*P*=0.03).

Plasma concentrations of oestradiol, progesterone, testosterone, SHBG, IGF-1 and IGFBP-3, obtained during the visit when the breast volumes were measured, were available for 139 women with data on breast volumes and CYP1A2*1F. In this subgroup, the interaction between coffee consumption and CYP1A2*1F genotype on breast volume was somewhat stronger (*β*=−0.341; *P*_interaction_=0.007) and became even stronger after further adjustment for hormone and growth factor levels (*β*=−0.401; *P*_interaction_=0.002).

Among current hormonal contraceptive users, coffee consumption was not associated with breast volume and there was no significant interaction between coffee and CYP1A2*1F on breast volume.

## Discussion

Our main finding was a significant interaction between coffee consumption, CYP1A2*1F genotype and breast volume among young healthy women who did not use hormonal contraceptives. This interaction was mainly driven by the fact that a moderate-to-high coffee intake was associated with lower breast volume in women with the C-allele. No association between coffee and breast volumes was observed in women with the CYP1A2*1F A/A genotype or in women who currently used hormonal contraception. As hormonal contraceptives lower the CYP1A2 enzyme activity (Rietveld *et al*, 1984), the latter observation was expected.

In this study, approximately 50% of the women carried the CYP1A2*1F A/A genotype, which is consistent with reported frequencies (Sachse *et al*, 1999; Nordmark *et al*, 2002). CYP1A2*1F is located in intron 1 and may not directly be associated with enzyme activity, but is rather linked to other polymorphisms with regulatory properties (Sachse *et al*, 1999). Coffee has been reported to predict 4% of the variation in CYP1A2 enzyme activity (Le Marchand *et al*, 1997).

In this study, breast volume was measured and approximated to a pyramid for practical reasons. A more exact procedure may have been to use water displacement, which we tried, but was impractical and less reproducible. Using bra cup size as a measurement of breast volume is less satisfactory, as cup-size labelling is not standardised (Ringberg *et al*, 2006).

Neither bra cup size nor breast volume reflects how much of the women's breast volume consists of breast parenchyma and how much is fat. A high BMI is positively correlated with larger cup sizes (Hall *et al*, 1999), which is in line with our findings. Others have reported that larger breasts consist of proportionally more fat and less parenchyma than smaller breasts in premenopausal women (Beijerinck *et al*, 1995; Kato *et al*, 1995). Most of our women were too young to have undergone mammography. We were thus unable to address whether coffee was associated with density, which is a stronger predictor of breast cancer risk than breast volume.

As coffee consumption was associated with lower breast volume among carriers of the C-allele with little effect among homozygous A/A carriers, mechanisms other than the induction of the CYP1A2 enzyme may be at play. Caffeine is the only major compound in coffee that is known to be metabolised by CYP1A2 and the effect could be attributed to the prolonged exposure of caffeine among C-allele carriers, as caffeine inhibits cell proliferation in mouse epidermal JB6 cells (Hashimoto *et al*, 2004).

Intake of caffeine has been positively associated with SHBG levels (Nagata *et al*, 1998) and inversely associated with bioavailable testosterone (Ferrini and Barrett-Connor, 1996), but CYP1A2*1F was not analysed. In this study, we did not observe any significant associations between coffee intake and hormone and growth factor levels. However, bioavailable testosterone levels were positively associated with breast volumes in both groups (data not shown). In contrast to our earlier study, we did not find that hormonal contraception significantly influenced breast volume (Jernström and Olsson, 1997). In this study, measured hormone levels did not explain the observed interaction between coffee and CYP1A2*1F on breast volume, although this became stronger after further adjustment for hormone levels.

In conclusion, the CYP1A2*1F genotype significantly modified the relationship between coffee consumption and breast volume in non-users of hormonal contraception. It is likely that various compounds in coffee exert a direct effect on the breast epithelium. As breast volume is associated with breast cancer risk in lean women, our finding is compatible with earlier reports of a protective effect of coffee on breast cancer risk restricted to women with the CYP1A2*1F C-allele.

## Figures and Tables

**Table 1:** Baseline characteristics of the 269 women included in the study

	**All women (*N*=269)**	CYP1A2*1F A/A (*N*=136)	CYP1A2*1F any C (*N*=131)
Median (inter quartile range) or %			
Year of birth	1970 (1965–1979)	1970 (1965–1975)	1970 (1964–1976)
Age at baseline (years)	29 (24–34)	29 (24–33)	30 (24–36)
Age at menarche (years)^a^	13 (12–13.4)	13 (12–13)	13 (12–14)
Age at first birth in parous women (years)	24.5 (22–28)	24 (23–27)	25 (22–28)
Nulliparous (%)	52	54	49
Ever smoker (%)^a^	42	41	42
Current smoker (%)^a^	23	24	22
Hormonal contraceptive use, ever (%)	92.5	93	92
Current hormonal contraceptive use (%)	42	48	36
			
*Daily coffee intake, cups (%)*			
None	(32.3)	(31.6)	(32.8)
<1.0	(3.0)	(2.9)	(3.1)
1.0–1.9	(5.9)	(7.6)	(4.6)
2.0–2.9	(17.1)	(16.9)	(16.8)
3.0–3.9	(6.7)	(5.1)	(8.4)
4.0–4.9	(11.5)	(15.4)	(7.6)
5.0–5.9	(5.6)	(4.4)	(6.9)
6+	(17.8)	(16.2)	(19.8)
			
Height (cm)^b^	168 (164–172)	168 (164–173)	168 (164–172)
Weight (kg)^b^	64 (58–74)	64 (58–75)	65 (58–73)
BMI (kg/m^2^)^b^	22.8 (20.9–25.6)	22.7 (20.8–26.2)	22.8 (20.9–24.9)
Waist-to-hip ratio^b^	0.76 (0.73–0.80)	0.77 (0.73–0.80)	0.75 (0.73–0.80)
Total breast volume (ml)^c^	756 (537–1101)	711 (555–1167)	777 (531–1040)

BMI=body mass index.

a One woman had not answered the question about age at menarche and the question about smoking.

b The body measurements were taken 5–10 days before the predicted onset of the next menstrual period, that is during cycle days 18–23 in most women.

c Total breast volumes were available for 255 women who were not currently breast-feeding or had undergone previous breast surgeries.

**Table 2:** *BRCA1/2* mutation status and CYP1A2*1F genotypes in the women included in the study. CYP1A2*1F genotypes were missing for two women from non-*BRCA1/2* families

	**All women *N*=269 N**	CYP1A2*1F A/A *N*=136 *N* (%)	CYP1A2*1F any C *N*=131 *N* (%)
*BRCA1*			
Carrier	29	11 (37.9)	18 (621)
Non-carrier	49	29 (59.2)	20 (40.8)
Not tested	16	8 (50.0)	8 (50.0)
			
*BRCA2*			
Carrier	7	4 (57.1)	3 (42.9)
Non-carrier	7	6 (85.7)	1 (14.3)
Not tested	4	3 (75.0)	1 (25.0)
			
Non-*BRCA1/2*	112	55 (50.0)	55 (50.0)
Untested families	45	20 (44.4)	25 (55.6)
